# *Francisella tularensis*: No Evidence for Transovarial Transmission in the Tularemia Tick Vectors *Dermacentor reticulatus* and *Ixodes ricinus*

**DOI:** 10.1371/journal.pone.0133593

**Published:** 2015-08-05

**Authors:** Marco Genchi, Paola Prati, Nadia Vicari, Andrea Manfredini, Luciano Sacchi, Emanuela Clementi, Claudio Bandi, Sara Epis, Massimo Fabbi

**Affiliations:** 1 National Reference Laboratory for Tularemia, Istituto Zooprofilattico Sperimentale della Lombardia e dell’Emilia Romagna “Bruno Ubertini”, Pavia, Italy; 2 Department of Biology and Biotechnology (Lazzaro Spallanzani), Università degli Studi di Pavia, Pavia, Italy; 3 Department of Veterinary Science and Public Health (Divet), Università degli Studi di Milano, Milan, Italy; The Johns Hopkins University School of Medicine, UNITED STATES

## Abstract

**Background:**

Tularemia is a zoonosis caused by the *Francisella tularensis*, a highly infectious Gram-negative coccobacillus. Due to easy dissemination, multiple routes of infection, high environmental contamination and morbidity and mortality rates, *Francisella* is considered a potential bioterrorism threat and classified as a category A select agent by the CDC. Tick bites are among the most prevalent modes of transmission, and ticks have been indicated as a possible reservoir, although their reservoir competence has yet to be defined. Tick-borne transmission of *F*. *tularensis* was recognized in 1923, and transstadial transmission has been demonstrated in several tick species. Studies on transovarial transmission, however, have reported conflicting results.

**Objective:**

The aim of this study was to evaluate the role of ticks as reservoirs for *Francisella*, assessing the transovarial transmission of *F*. *tularensis* subsp. *holarctica* in ticks, using experimentally-infected females of *Dermacentor reticulatus* and *Ixodes ricinus*.

**Results:**

Transmission electron microscopy and fluorescence in situ hybridization showed *F*. *tularensis* within oocytes. However, cultures and bioassays of eggs and larvae were negative; in addition, microscopy techniques revealed bacterial degeneration/death in the oocytes.

**Conclusions:**

These results suggest that bacterial death might occur in oocytes, preventing the transovarial transmission of *Francisella*. We can speculate that *Francisella* does not have a defined reservoir, but that rather various biological niches (e.g. ticks, rodents), that allow the bacterium to persist in the environment. Our results, suggesting that ticks are not competent for the bacterium vertical transmission, are congruent with this view.

## Introduction

Tularemia is a zoonosis caused by *Francisella tularensis*, a highly infectious Gram-negative coccobacillus which has been isolated from over 250 species of mammals, birds, reptiles, amphibians, fish and invertebrates [1]. *F*. *tularensis* can be transmitted by several routes, including direct contact with infected blood and tissues through wounds, intact skin and mucous membranes, ingestion of contaminated food or water, inhalation of aerosols and arthropod bites [1]. Two *F*. *tularensis* subspecies are most often associated with human and animal disease: *F*. *tularensis* subsp. *tularensis* (type A), found only in North America, and *F*. *tularensis* subsp. *holarctica* (type B), found throughout the Northern Hemisphere. Due to easy dissemination, multiple routes of infection, high environmental contamination and morbidity and mortality rates, *F*. *tularensis* is considered a potential bioterrorism threat and classified as a category A select agent by the Centers for Disease Control (CDC, Atlanta, Georgia, USA). For this reason, several studies have been carried out to evaluate variability of clinical features, feasibility and options for mass prophylaxis (i.e. vaccine), therapeutic approaches for treatment, development of improved diagnostic tests, including genome sequencing of several strains [2]. However, the ecology of *F*. *tularensis* and how the bacterium persists between outbreaks is not clear, and, in particular, the actual reservoir(s) has not yet been uncovered.

Arthropods, especially ixodid ticks, are considered to play a prominent role in enzootic persistence of *F*. *tularensis* infections in nature and their role as potential vectors to humans and animals has been shown [3,4]. Tick bites are among the most prevalent modes of transmission in North America, where the tick species most often associated with human infections are *Dermacentor andersoni*, *D*. *variabilis*, and *Amblyomma americanum* [3,5]. In Europe, the percentage of tularemia patients developing the disease after tick bite varies from 13% to 26% [6,7]. *Ixodes ricinus* and *D*. *reticulatus* are the species most frequently involved, with variable infection prevalences, e.g. from ~ 2 to ~ 4% [8,9]. Furthermore, ticks have been indicated as a possible reservoir [10–12], although their reservoir competence has yet to be defined. In fact, whereas the transstadial (larva—nymph—adult) transmission has been demonstrated in different hard tick species [4,13,14], transovarial transmission (adult—egg) is still widely debated. The studies carried out until now have produced contradictory results. Earlier studies in fact demonstrated transovarial transmission in several species of ixodid ticks (e.g. *D*. *andersoni* and *D*. *variabilis*) [15–18], whereas more recent experiments failed to confirm the transmission of *F*. *tularensis* to progeny of infected ticks (e.g. *D*. *andersoni*, *D*. *variabilis* and *I*. *ovatus*) [19–22].

The aim of our study was to verify transovarial transmission of *F*. *tularensis* subsp. *holarctica* by experimentally-infected female *D*. *reticulatus* and *I*. *ricinus* ticks. Experimental exposure and transmission were investigated using a fully virulent *F*. *tularensis* subsp. *holarctica* strain. Moreover, in order to mimic the natural conditions of infection and interaction among vector, bacterium and host, we used ticks fed on bacteremic animals.

## Materials and Methods

### Tick collection

Unfed questing adult *D*. *reticulatus* and *I*. *ricinus* were collected by flagging from March to September 2013 on private land of the Province of Pavia, North Italy (44°59'12.3"N, 9°11'10.7"E), upon agreement with the owner of the land. Ticks were identified using taxonomic keys for adults [23,24], and stored in a vial at +4°C for 10–14 days until testing. To assessed the presence of *F*. *tularensis* and *F*. *tularensis*-like endosymbionts, about 20 *D*. *reticulatus* female ticks and 20 *I*. *ricinus* female ticks were processed by real-time PCR.

### Ethics

The study was designed and carried out in strict accordance with principles of the 3Rs, European directive (2010/63/EU), National Animal Testing Rules (D.Lgs 116/92). The study was authorized by the Italian Ministry of Health (authorization no. 15981 of 20/06/2012). The study was approved before the Istituto Zooprofilattico Sperimentale della Lombardia e dell'Emilia Romagna (IZSLER) had adopted its own Ethics Committee. Based on previous reports and 3Rs we have chosen to use the guinea pigs for the following reasons: i) rabbits do not develop the disease; ii) hares are difficult to handle and hold in a cage. Laboratory animals were provided by the authorized animal facility IZSLER—Brescia and all efforts were made to minimize animal suffering. In agreement with European directive (2010/63/EU), all animals used in the present research, before being sacrificed by isofluorane overdose were anesthetized with ketamine (500 μg/kg IM). All procedures that required handling of live *F*. *tularensis* were carried out at biosafety level 3.

### Guinea pig tick infestation

The experimental design was performed in 6 replicates. For each replicate, 2 guinea pigs, aged approximately 15 weeks, were used. Animals were placed in individual cages and fed ad libitum. Overall, 150 female and 210 male of *D*. *reticulatus* and 150 female and 210 male of *I*. *ricinus* adult ticks were used. Replicates were identified as A (1–6) for *D*. *reticulatus*, and B (1–6) for *I*. *ricinus*.

**Fig 1:**
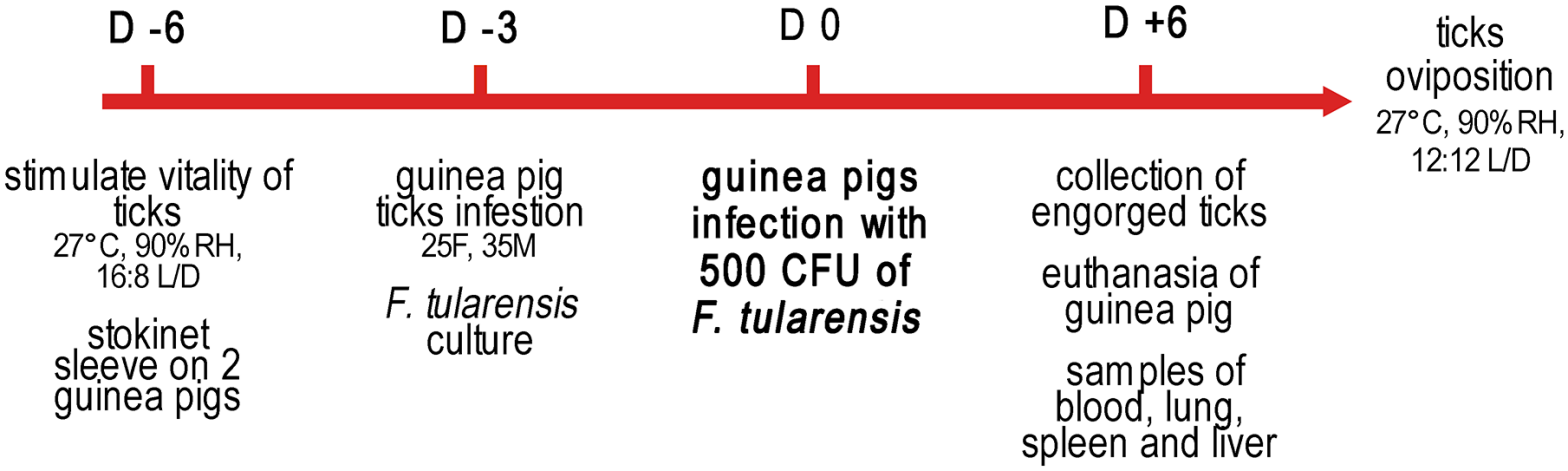
Experimental design. Guinea pig infection and tick blood meal.

On Day -6 (Fig 1) ticks were placed inside a humid glass container in an incubator at 27°C, with 90% relative humidity (RH) and light-dark (L/D) cycle of 16:8 hours, in order to stimulate vitality.

For each replica, two guinea pigs, aged approximately 15 weeks, were prepared by shaving a circular area of approximately 8 cm in diameter, from the shoulder blades to the mid-thorax. A cotton stockinette sleeve (4.5 cm diameter and 10 cm long) was fixed to the area with non-toxic glue (Kamar spare glue; Kamar, Inc, CO 80477). On Day -3, 25 female ticks and 35 male ticks for each guinea pig were placed inside the stockinette. The presence of a certain number of male ticks is critical for female fecundation, and for the completion of an adequate blood meal.

### Pilot study and inoculum

The culture strain 21851/2006 (Fth25) of *F*. *tularensis* subsp. *holarctica*, isolated in Italy from a dead hare, was used to prepare the inoculum. Two pilot studies were performed. The first was carried out on no-infected guinea pigs to assess the time needed to complete the tick blood meal of both tick species (*I*. *ricinus* and *D*. *reticulatus*). The second pilot study was conducted to evaluate the number of colony-forming units (CFU)/ml to be used in the inoculum that would keep the guinea pigs alive for 6 days, in order to allow enough time for ticks to complete their blood meal. Based on previous reports [16,25] and on the experience of our National Reference Laboratory for Tularemia, three concentrations were tested: 200 CFU, 400 CFU, and 500 CFU. The guinea pig blood samples after their death were immediately plated to assess bacteremia.

*F*. *tularensis* subsp. *holarctica* strain was grown on cysteine heart agar (CHA) containing 8% horse blood at 37°C for 72 hours. After, on Day -3, bacteria were suspended in sterile saline solution and diluted to a final concentration of ~1.65 × 10^3^ CFU/ml. A target inoculating dose of 500 CFU was selected based on the previous pilot study. The concentrations of the inocula were confirmed by plating bacteria in duplicate on CHA and counting colony forming units after 72 hours of incubation at 37°C. On Day 0, the tick-infested guinea pigs were inoculated subcutaneously with 500 CFU suspended in 0.3 ml of sterile saline solution.

### Evaluation of transmission to ticks and eggs

Six days post-infection, engorged ticks were collected from the stockinette, placed individually in vials and incubated at 27°C, 90% RH and 12:12 L/D until completion of oviposition. A pool of tick faeces was also collected from the stockinette, and culture and real-time PCR were carried out. Subsequently, guinea pigs were humanely euthanized by isofluorane overdose and organs were aseptically removed to assess pathological lesions. Livers, spleens and lungs were homogenized (1:1 w/v) in sterile saline and processed by real-time PCR and culture. Blood samples were collected by cardiac puncture to determine CFU counts.

**Fig 2:**
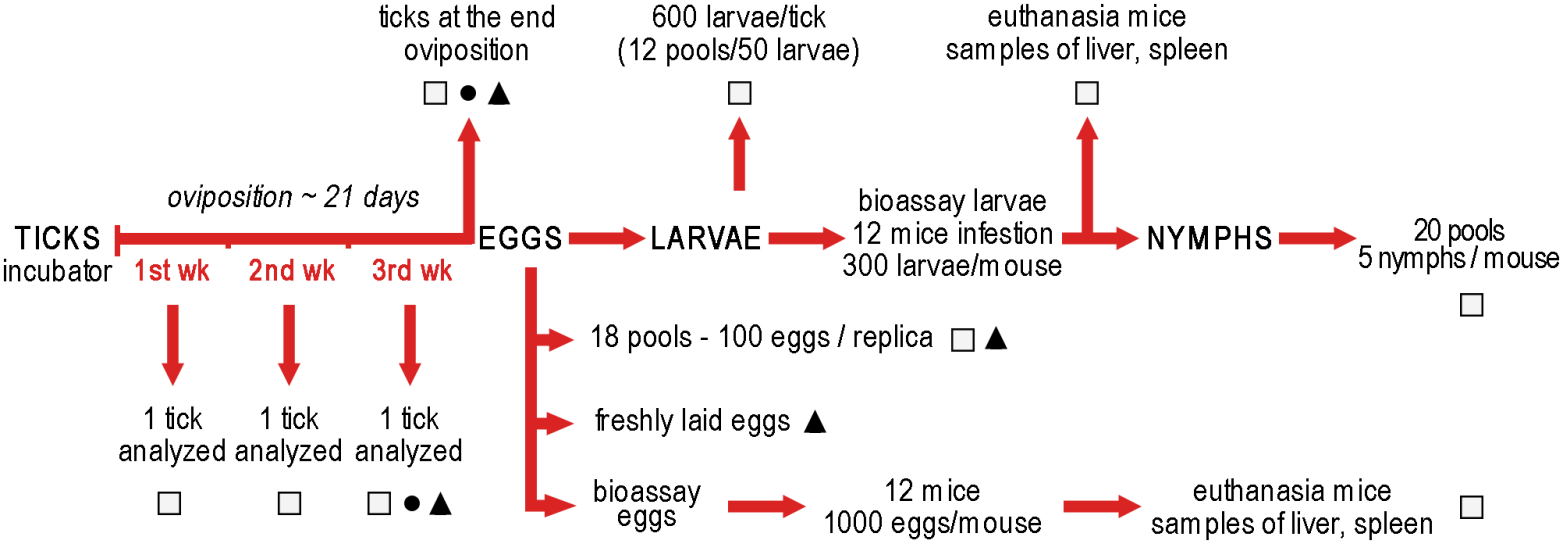
Experimental design: moulting and testing samples. Real-time PCR and culture: □; Transmission Electron Microscopy: ●; Fluorescence In Situ Hybridization: ▲.

To confirm that engorged and egg-laying ticks acquired and maintained the infection, a single tick was analyzed after 7, 14 and 21 days for each guinea pig (a total of 36 ticks). Each tick was placed in a mortar under sterile conditions and triturated in 1 ml of saline solution. The filtered suspension was cultured and analyzed by real-time PCR. In addition, the ovaries and salivary glands of 12 ticks, collected at day 21 for each guinea pig, were placed in a saline solution and divided into two pieces for transmission electron microscopy (TEM) and fluorescence in situ hybridization (FISH) (Fig 2).

### Evaluation of transmission to larvae and nymphs

At the end of laying, eggs from each tick were collected and placed in 50 ml tubes in an incubator at 24°C and 90% RH to obtain larvae. Eighteen pools of 100 eggs for each replica were obtained and processed by real-time PCR and culture. To exclude surface contamination with *F*. *tularensis*, eggs were washed six times in 200 μl DEPC-PBS. The first and last wash were processed by real-time PCR (50 μl) and culture (150 μl). Eggs were crushed in a microcentrifuge tube using an insulin needle before real-time PCR and culture assays. Eggs for FISH analysis were collected after removal of all eggs laid in the first two days. In this way, oviposition and collection of fresh eggs was assured. In addition, a bioassay was carried out to verify the viability of *F*. *tularensis* from the eggs. Briefly, six pools of approximately 2,000 eggs each were obtained from eggs laid by 9–10 ticks for each replica. Eggs were sampled from different points of egg packs. Subsequently eggs were crushed and homogenized with 0.5 ml saline solution, the homogenate was separated in 2 aliquots of 0.25 ml and inoculated subcutaneously in 2 female mice. Each pair of mice (12 mice in total) were placed in separate cages, fed ad libitum and observed daily for one month. Subsequently, mice were humanely euthanized by isofluorane overdose and the livers and spleens were processed by real-time PCR and culture.

After hatching of tick eggs, approximately 600 larvae (12 pools of 50 larvae) for each oviposition were processed by real-time PCR and culture. A bioassay was also carried out with 12 female mice. Mice were placed in individual cages, infested with 300 larvae using larvae laid by 10 tick-progenitors for each replica and observed for one month. To prevent the escape of larvae, the cage was put inside a water-filled tray and a double-sided tape was attached along the edge of the container. The bottom cage was covered with paper towels to facilitate the collection of engorged larvae. Eight times per day, paper towels, walls of the cage, water and double-sided tape, were checked for the presence of engorged larvae. After one month, mice were euthanized and their organs were processed by real-time PCR and culture.

Engorged larvae were placed in 50 ml vial and stored in an incubator at 24°C and 90% RH for moulting. Subsequently, nymphs were collected. Twenty pools of 5 nymphs for each infested mouse were analyzed by real-time PCR and culture.

All female ticks at the end of the oviposition were dissected and their salivary glands, midgut and ovary were analyzed by PCR, TEM, FISH and culture.

### Real-time PCR

The surface of the ticks was first disinfected by washing three times in 70% ethanol, rinsed in sterile distilled water and dried at room temperature. Ticks, eggs and faeces were homogenised by mechanical agitation in a TissueLyser II (Qiagen, Milan, IT) and total DNA was purified using the DNeasy Blood and Tissue Kit (Qiagen, Milan, IT) according to the manufacturer’s instructions. Purified DNA was eluted in 200 μl AE buffer for adult ticks and 50 μl AE for nymphs and eggs. Total DNA from guinea pig blood and organs was extracted with the same kit. Detection of *F*. *tularensis* DNA was performed in CFX-96 Real-Time system (Biorad, Milan, IT) by real-time PCR targeting the 23kDa gene, as described [26]. Negative (no template) and positive (DNA of *F*. *tularensis* subsp. *tularensis* strain ATCC 6223) controls were included in each run, and an internal control was included in each sample (Taqman Exogenous Internal Positive Control, AB Applied Biosystems).

### Transmission Electron Microscopy (TEM)

Tick samples were harvested and prefixed in Karnowsky’s fixative in cacodylate buffer (pH 7.2). After post-fixation in 2% OsO4 in 0.1 M cacodylate buffer for 1.5 hours at 4°C, samples were washed in corresponding buffer, dehydrated in ethanol series, transferred to propylene oxide and embedded in Epon 812. Semi-thin sections were stained with 0.05% toluidine blue in 1% sodium tetraborate and examined by optical microscopy. Thin sections (80 nm) were stained with saturated uranyl acetate, followed by Reynolds lead citrate and examined with Zeiss EM900 transmission electron microscope at 80 kV.

### Fluorescence In Situ Hybridization (FISH)

For FISH assays, we utilized the 23S rRNA universal probe for *F*. *tularensis* W_all1448CY5 (CAACCATTCGCCAGGCCT labelled at the 5’ with the fluorochrome Cy5, absorption and emission at 650 nm and 670 nm, respectively) [27]. An additional probe, EUB338CY3 (GCTGCCTCCCGTAGGAGT labelled at the 5’ with the fluorochrome Cy3, absorption and emission at 550 nm and 570 nm, respectively), routinely used as a universal bacterial probe, was used as bacterial positive control [28]. The probes non-EUB338 and non-W_all1448CY5, which are the complementary (antisense) sequence respectively to probe EUB338CY3 and W_all1448CY5, served as a negative control for nonspecific binding. FISH assays were performed following the protocol published in [29].

Briefly, all dissected samples were fixed in 4% formaldehyde buffered with PBS for 30 min RT. Pre-treatment with proteinase K (0.5 μg/ml) was carried out before hybridization. Samples were washed twice in a solution of 1X PBS containing 1% Tween 20 and once in 1X PBS for 5 min at RT. Hybridization was carried out in the dark for 2 h at 46°C, with 20 μl of hybridization buffer (10X SSC, 35% formamide, Denhardt's solution 0.5%, 30 ng ml^-1^ probes). After hybridization, tick samples were washed in 500 μl of washing buffer 2X SSC for 10 min and 500 μl of 0.1X SSC for 10 min. Samples were then mounted on glasses slides with 40 μl 1,4-diazabicyclo[2.2.2]octane (DABCO) and observed using a laser-scanning confocal microscope (Leica, Wetzlar, Germany).

Fresh eggs of ticks were instead processed as follows. Before fixation, eggs were vortexed for 2 to 3 min to clean their surface, and washed three times with 1X PBS. Eggs were fixed in 3% formaldehyde buffered with PBS incubated at 4°C for 6h and washed three times in 1X PBS. Eggs were immobilized on 10-well microscope slides with a drop of 0.5% Ultra Pure Low Melting Point Agarose (Life Technologies, Carlsbad, California) and crushed to allow penetration of the probes into the cytoplasm during hybridization. The hybridization was carried out as described above using the probes W_all1448CY5 and Apis2Pa (CCTCTTTGGGTAGATCC, labelled at the 5’ with the fluorochrome FAM), as gammaproteobacteria universal probe. In addition, 75 ng/ml of 4′,6-diamidino-2-phenylindole (DAPI) were added for nuclei detection and incubated for 15 min at RT.

## Results

All the 40 female ticks processed by PCR to assess the presence of field strains of *F*. *tularensis* and *F*. *tularensis*-like endosymbionts resulted negative.

Typical macroscopic lesions of tularemia were observed at necropsy of euthanized guinea pigs. Bacterial culture and real-time PCR confirmed the infection by *F*. *tularensis* subsp. *holarctica* in all 12 animals form the 6 replicates. The bacterial load in blood samples attained an average of 1,120 CFU/ml (range: 914–1,350 CFU/ml).

Tables 1 and 2 summarize the total counts of recovered engorged ticks, mortality and oviposition of *D*. *reticulatus* and *I*. *ricinus*. One hundred and nine *D*. *reticulatus* adult females out of 150 (72.7%) used in the study completed the blood meal; 18 of these were analyzed during the oviposition. Of the 91 remaining engorged ticks, 13 (14.3%) died during the oviposition, 14 (15.4%) did not depose and 64 (70.3%) completed the oviposition. One hundred and fifty *I*. *ricinus* adult females out 133 (88.6%) completed the blood meal; 18 of these were analyzed during the oviposition. Of the 115 remaining engorged ticks, 5 (4.3%) died during the oviposition, 15 (13.0%) did not depose and 95 (82.6%) completed the oviposition.

**Table 1:** Engorged *Dermacentor reticulatus* female ticks after feeding on guinea pigs infected with *F*. *tularensis* subsp. *holarctica*.

Ticks	Replicates on guinea pigs						total
	A1*	A2	A3	A4	A5	A6	
**initial no. females**	25	25	25	25	25	25	150
**engorged**	18	16	20	21	15	19	109 (72.7%)
**examined during oviposition**	3	3	3	3	3	3	18
**dead during oviposition**	3	3	2	2	2	1	13 (14.3%)
**no oviposition**	2	2	4	2	1	3	14 (15.4%)
**total completing oviposition**	10	8	11	14	9	12	64 (70.3%)

**Table 2:** Engorged *Ixodes ricinus* female ticks after feeding on guinea pigs infected with *F*. *tularensis* subsp. *holarctica*.

Ticks	Replicates on guinea pigs						total
	B1	B2	B3	B4	B5	B6	
**initial no. females**	25	25	25	25	25	25	150
**engorged**	23	22	19	21	25	23	133 (88.6%)
**examined during oviposition**	3	3	3	3	3	3	18
**dead during oviposition**	0	1	1	2	0	1	5 (4.3%)
**no oviposition**	2	3	1	1	5	3	15 (13.0%)
**total completing oviposition**	18	15	14	15	17	16	95 (82.6%)

All ticks of both species, *D*. *reticulatus* and *I*. *ricinus*, examined during and/or at the end of oviposition were positive by real-time PCR and culture. Eleven pools of tick faeces collected from the stockinettes after the infected blood meal were PCR positive, but resulted negative at culture. The A5 faeces pool (from *D*. *reticulatus*) was negative for PCR and culture.

**Fig 3:**
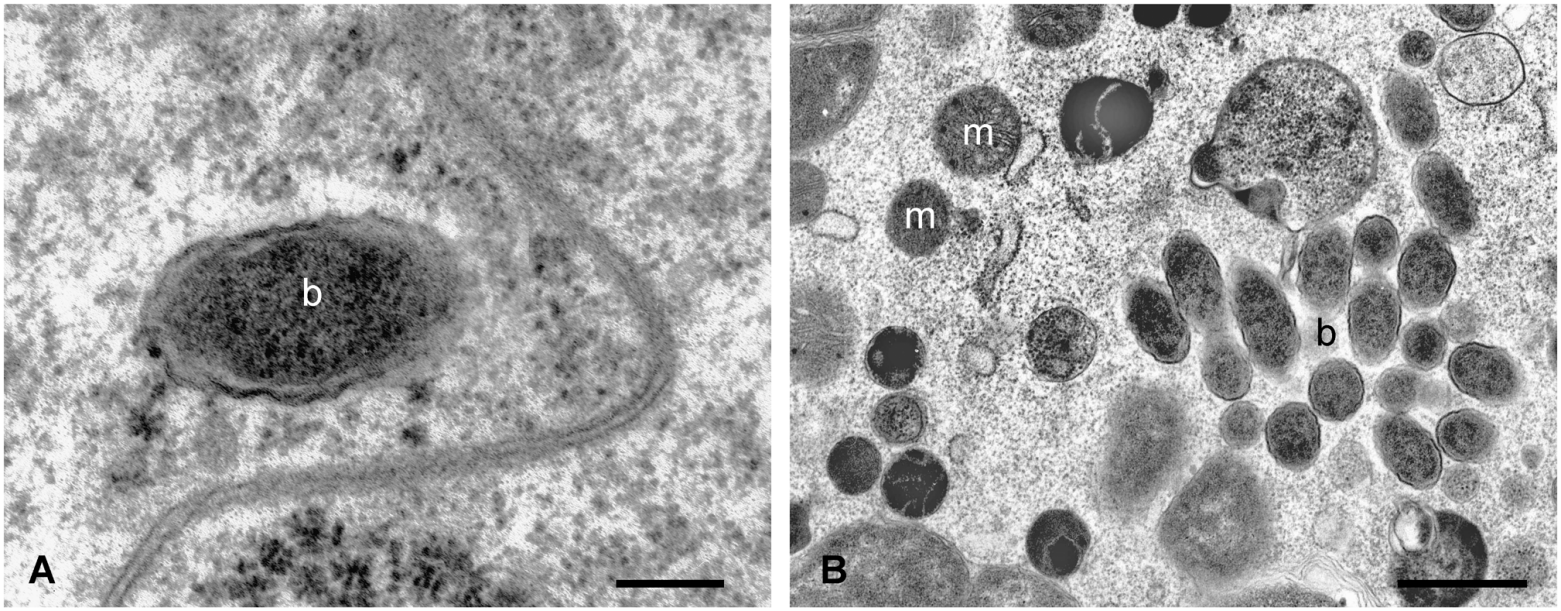
TEM micrographs of *Ixodes ricinus* pre-vitellogenic oocyte after feeding on guinea pigs infected with *F*. *tularensis* subsp. *holarctica*. The morphology of some inclusions is typical of a Gram-negative bacterium, and their size is congruent with that of bacteria from the *Francisella* genus. m: mithocondrium, b: bacterium. Scale bar: A 0.22 μm, B 1.1 μm.

TEM examination of *D*. *reticulatus* and *I*. *ricinus* ovaries during and at the end of oviposition showed that the oocytes contain Gram-negative coccobacillar bacteria of approximately 0.4 μm, referable to *F*. *tularensis* (Fig 3).

**Fig 4:**
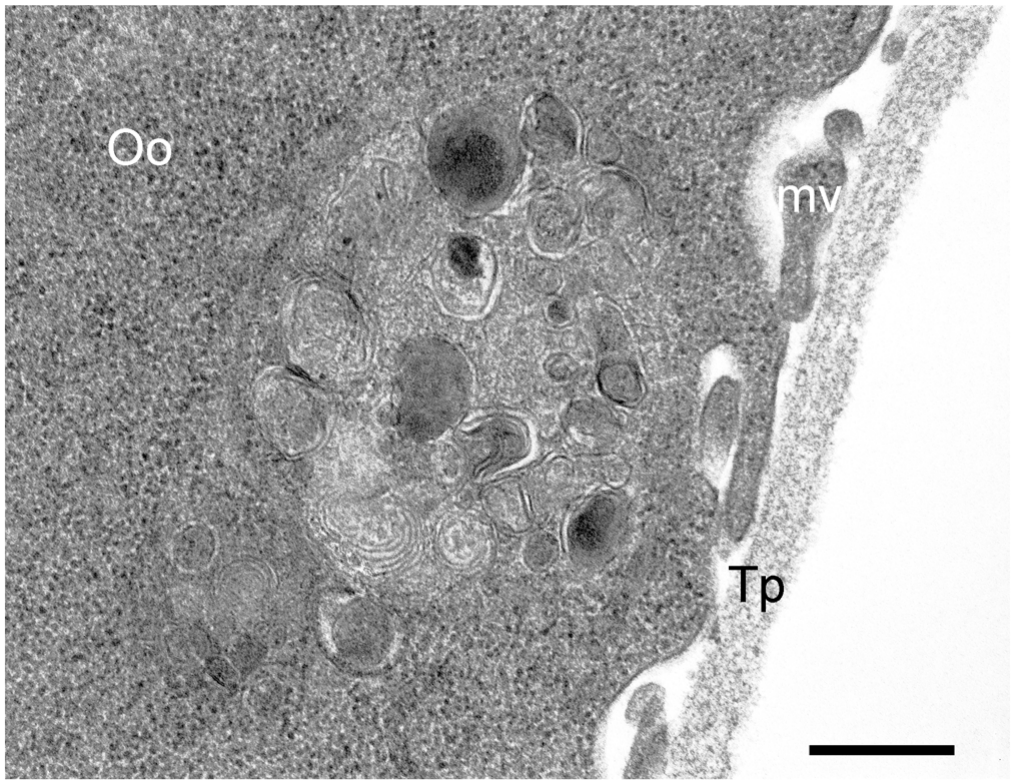
TEM micrographs of *Ixodes ricinus* pre-vitellogenic oocyte after feeding on guinea pigs infected with *F*. *tularensis* subsp. *holarctica*. Note the presence of residual bodies into the large, phagocytic vacuole. Oo: oocyte, mv: microvilli, Tp: tunica propria. Scale bar: 0.37 μm.

In addition to free bacteria in the oocyte cytoplasm, phagocytic vacuoles containing residual bacterial bodies were observed (Fig 4).

**Fig 5:**
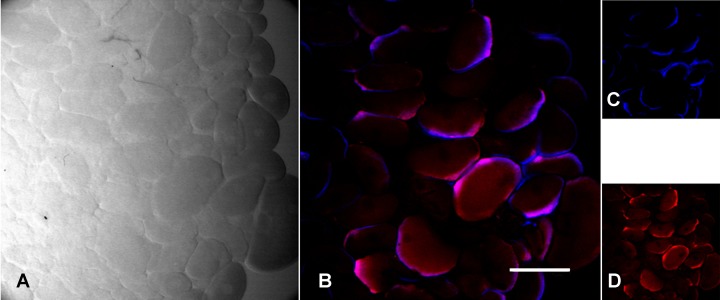
FISH staining with a probe specific for *Francisella tularensis* in *Dermacentor reticulatus* oocytes after feeding on guinea pigs infected with *F*. *tularensis* subsp. *holarctica*. (A) Image obtained from light transmission; (B) Overlay image with blue signal for *F*. *tularensis* (23S rRNA probe for *F*. *tularensis* labelled with the fluorochrome Cy5) and red signal for universal eubacterial probe EUB338; (C) 23S rRNA probe for all *F*. *tularensis*; (D) universal eubacterial probe EUB338. Scale bar: 200 μm.

**Fig 6:**
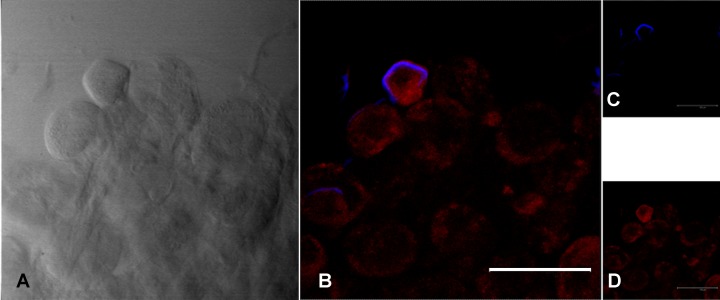
FISH staining with a probe specific for *Francisella tularensis* in *Ixodes ricinus* salivary glands from female after feeding on guinea pigs infected with *F*. *tularensis* subsp. *holarctica*. (A) Image obtained from light transmission; (B) Overlay image with blue signal for *F*. *tularensis* (23S rRNA probe for *F*. *tularensis* labelled with the fluorochrome Cy5) and red signal for universal eubacterial probe EUB338; (C) 23S rRNA probe for *F*. *tularensis*; (D) universal eubacterial probe EUB338. Scale bar: 100 μm.

In both tick species, FISH staining using the probe specific for *F*. *tularensis* revealed brightly stained bodies in in the oocytes, but also in the salivary glands (Figs 5 and 6).

FISH-stained bodies were particularly concentrated around the periphery of the oocytes.

The solutions used to wash *D*. *reticulatus* and *I*. *ricinus* eggs were negative by both PCR and culture. Conversely, PCR was positive on 18 pools of 100 eggs, yet culture was negative. Freshly laid eggs of both tick species were also negative by FISH. Bioassay performed by the inoculum of 1,000 homogenated eggs in each of 12 mice (0.25 ml/ mouse, SC) was negative during the whole observation period for both tick species. At 30 days post-inoculation, no pathological signs were observed and PCR and cultures were negative in all mice organs.

For both tick species, the PCR carried out on the larvae form the same progenitor-ticks were in all cases positive to *F*. *tularensis* (12 pools of 50 larvae per tick), whereas culture was consistently negative from the larvae. All of the 12 mice infected with 300 larvae survived with no signs of disease, and their organs were negative by PCR and culture. After moulting, all examined pools of *D*. *reticulatus* and *I*. *ricinus* nymphs were negative at PCR.

## Discussion

A limited number of studies have investigated the transovarial transmission of *F*. *tularensis* in ticks. All studies were published between 1920 and 1960 when molecular techniques had not yet been developed and no *F*. *tularensis* subspecies had yet been defined [30]. In 1926, for the first time, Parker and Spencer [15] demonstrated the transovarial transmission of *F*. *tularensis* from infected *D*. *andersoni* ticks and later in *D*. *variabilis* [16–18]. On the other hand, several studies failed to confirm the transovarial passage of this bacterium to the progeny [19–22].

Our study provides no evidence for the transovarial transmission of *F*. *tularensis* subsp. *holarctica* in *D*. *reticulatus* and *I*. *ricinus*, the most important vectors of *F*. *tularensis* in Europe. This is the first study to assess the role of *D*. *reticulatus* and *I*. *ricinus* as a reservoir of *Francisella* with a known *Francisella* subspecies and with defined reproducible bacterial inocula. Moreover, we attempted to replicate the natural infection route, considering that host-bacteria-vector interaction may be of importance. This method allowed the dissemination of bacteria to all tick tissues, including the ovaries, in a "natural way". In fact, according to Horzempa et al [31], microarray analysis of *F*. *tularensis* following a shift from 26°C (environmental) to 37°C (mammalian) has shown that 11% of the bacterial genes are differentially-regulated and 40% of the protein-coding genes induced at 37°C are implicated in virulence or intracellular growth of *Francisella*. Furthermore, in experimental infections where sheep-fed *D*. *variabilis* were inoculated into the hemocele with the *F*. *tularensis* subsp. *holarctica* live vaccine strain, bacteria were observed exclusively in the tick oocyte shell and tunica propria, but not within the oocyte cytoplasm [32]. In summary, we believe that the infection of ticks after feeding on an infected host is required to ensure a proper colonization of the arthropod.

The ecology and biology of *Francisella* spp. has been studied in recent years, but the actual reservoir of infection has yet to be clarified. In our study, real-time PCR and culture showed that infection was maintained in both tick species *D*. *reticulatus* and *I*. *ricinus* from the end of tick feeding until oviposition, with colonization of the ovary and the salivary glands. Furthermore, in all egg pools and larvae from infected ticks, *Francisella*-DNA was detected by real-time PCR. However, although TEM and FISH showed *F*. *tularensis* dissemination into ovaries, with bacteria within the oocytes, the negative findings in FISH of eggs and the negative cultures and bioassays with eggs and larvae, suggest that *Francisella* death occurs in pre-vitellogenic oocytes, before eggs are laid; bacterial remnants, i.e. DNA, are thus likely maintained in the developing individuals,up to larvae. This interpretation is supported by transmission electron microscopy, that showed vacuoles in the oocytes containing degenerating Gram-negative bacteria, referable to *F*. *tularensis* in size and shape. It is worth noting that bioassay has a sensitivity below 10 CFU, which is possibly higher than the sensitivity of the real-time PCR protocol used in this study [26].

Our results suggest that transmission of *F*. *tularensis* from infected female ticks to their progeny does not occur, or is very rare. When considered in conjunction with the negative fitness of *Francisella*-infected ticks, whose survival rate is impaired, repletion time increases and moult efficiency diminishes [4,16,33], our results suggest that ticks are unlikely to represent a true reservoir for *F*. *tularensis*. Nonetheless, as recent studies have also shown, we should consider that different subpopulations of *F*. *tularensis* subspecies display behavioral differences in geographical distribution, virulence and animal hosts [34], which could also influence their role as reservoir. For instance, significantly higher mortality was observed between *F*. *tularensis* type A1b infections (24%) compared with A1a (4%) and A2 (0%) [35]. Furthermore, different tick species may be more or less competent reservoirs for different pathogens [36]. In our study we used a virulent strain of *F*. *tularensis* subsp. *holarctica*, infecting ticks of the species *D*. *reticulatus* and *I*. *ricinus*. For these reasons, although we believe it unlikely, we cannot exclude the possibility that a different transmission pattern could be observed in other species of ticks and/or with different subspecies or subpopulations of *F*. *tularensis*.

In conclusion, even though our observations do not support the possibility that *D*. *reticulatus* and *I*. *ricinus* are a true *reservoir* for *F*. *tularensis*, these arthropods will continue to play an important role in the ecology of the disease. In fact, ticks can both transmit the bacteria to two potential hosts through their stage-to-stage transmission [4,16,30] and maintain the organism in the environment for extended periods. *F*. *tularensis* can in fact survive up to 710 days in *D*. *marginatus* [20]. Furthermore, *F*. *tularensis* is able to replicate in the salivary glands of tick [32], and tick saliva can then facilitate the infection of the host, thanks to its immunomodulatory properties [37]. Moreover *F*. *tularensis* can be harboured in the faeces of infected ticks [4,38], as shown also by our results. Therefore, even in the absence of a transovarial transmission, ticks are able to maintain the infection in the environment during the inter-epizootic period and can be identified as reservoirs *de facto* [12], or long-term reservoirs [11], or more properly, in our opinion, "long-term vectors" of *F*. *tularensis*.

Based on our results, we can speculate that *F*. *tularensis* does not have a defined reservoir, but that rather various biological niches allow the persistence of bacteria in the environment, including rodents and lagomorphs, amoebae [39], mosquitoes [40] and ticks. The multiple host species that are susceptible to infection, along with the different routes of transmission, capability of biofilm formation [41], and its ability to survive in the environment for long periods all add up to efficient transmission of *F*. *tularensis*.

## Supporting Information

S1 Checklist This experiment was performed in accordance with the NC3Rs ARRIVE Guidelines for Reporting Animal Research. Kilkenny C, Browne WJ, Cuthill IC, Emerson M, Altman DG (2010) Improving bioscience research reporting: the ARRIVE guidelines for reporting animal research. PLoS Biol 8: e1000412.
(PDF)

S1 Fig FISH staining with specific probe for *Francisella tularensis* in cytoplasm of eggs of *Dermacentor reticulatus* after ovipostition. (A) Cellular nuclei stained for cell viability with DAPI (blue) and (B) negative signal after hybridisation with *F*. *tularensis 23S rRNA probe labelled with the fluorochrome Cy5*.
(TIF)

S2 Fig FISH staining with specific probe for *Francisella tularensis* in cytoplasm of eggs of *Ixodes ricinus* after oviposition. (A) Cellular nuclei stained for cell viability with DAPI (blue) and (B) negative signal after hybridisation with F. tularensis 23S rRNA probe labelled with the fluorochrome Cy5.
(TIF)

S3 Fig FISH staining with specific probe for *Francisella tularensis* in *Ixodes ricinus* oocytes after feeding on guinea pigs infected with *F*. *tularensis* subsp. *holarctica*. (A) Image obtained from light transmission; (B) Overlay image with blue signal for *F*. *tularensis (23S rRNA probe for F*. *tularensis labelled with the fluorochrome Cy5)* and red signal for universal eubacterial probe EUB338; (C) 23S rRNA probe for all *F*. *tularensis*; (D) universal eubacterial probe EUB338. Scale bar: 200 μm.
(TIF)

S4 Fig FISH staining with a probe specific for *Francisella tularensis* in *Dermacentor reticulatus* salivary glands from female after feeding on guinea pigs infected with *F*. *tularensis* subsp. *holarctica*. (A) Image obtained from light transmission; (B) Overlay image with blue signal for *F*. *tularensis (23S rRNA probe for F*. *tularensis labelled with the fluorochrome Cy5)* and red signal for universal eubacterial probe EUB338; (C) 23S rRNA probe for *F*. *tularensis*; (D) universal eubacterial probe EUB338. Scale bar: 100 μm.
(TIF)
