# Supplementary figures and images for: Francisella tularensis: No Evidence for Transovarial Transmission in the Tularemia Tick Vectors Dermacentor reticulatus and Ixodes ricinus
Source: PLoS One. 2015 Aug 5;10(8):e0133593. doi: 10.1371/journal.pone.0133593 (PMC4526560; doi:10.1371/journal.pone.0133593)

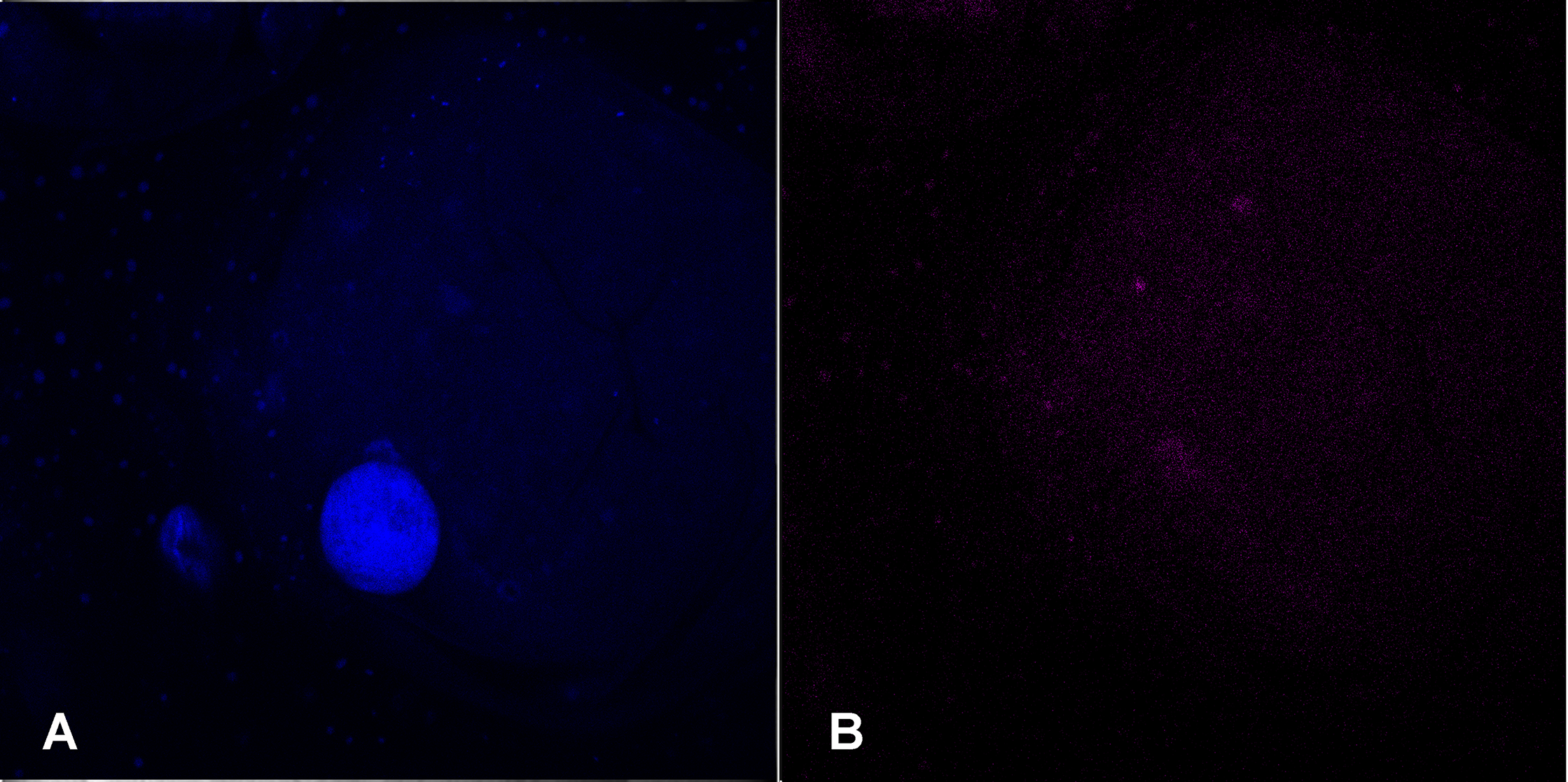

Supplement: S1 Fig — (A) Cellular nuclei stained for cell viability with DAPI (blue) and (B) negative signal after hybridisation with F. tularensis 23S rRNA probe labelled with the fluorochrome Cy5. (TIF) [file pone.0133593.s002.tif]

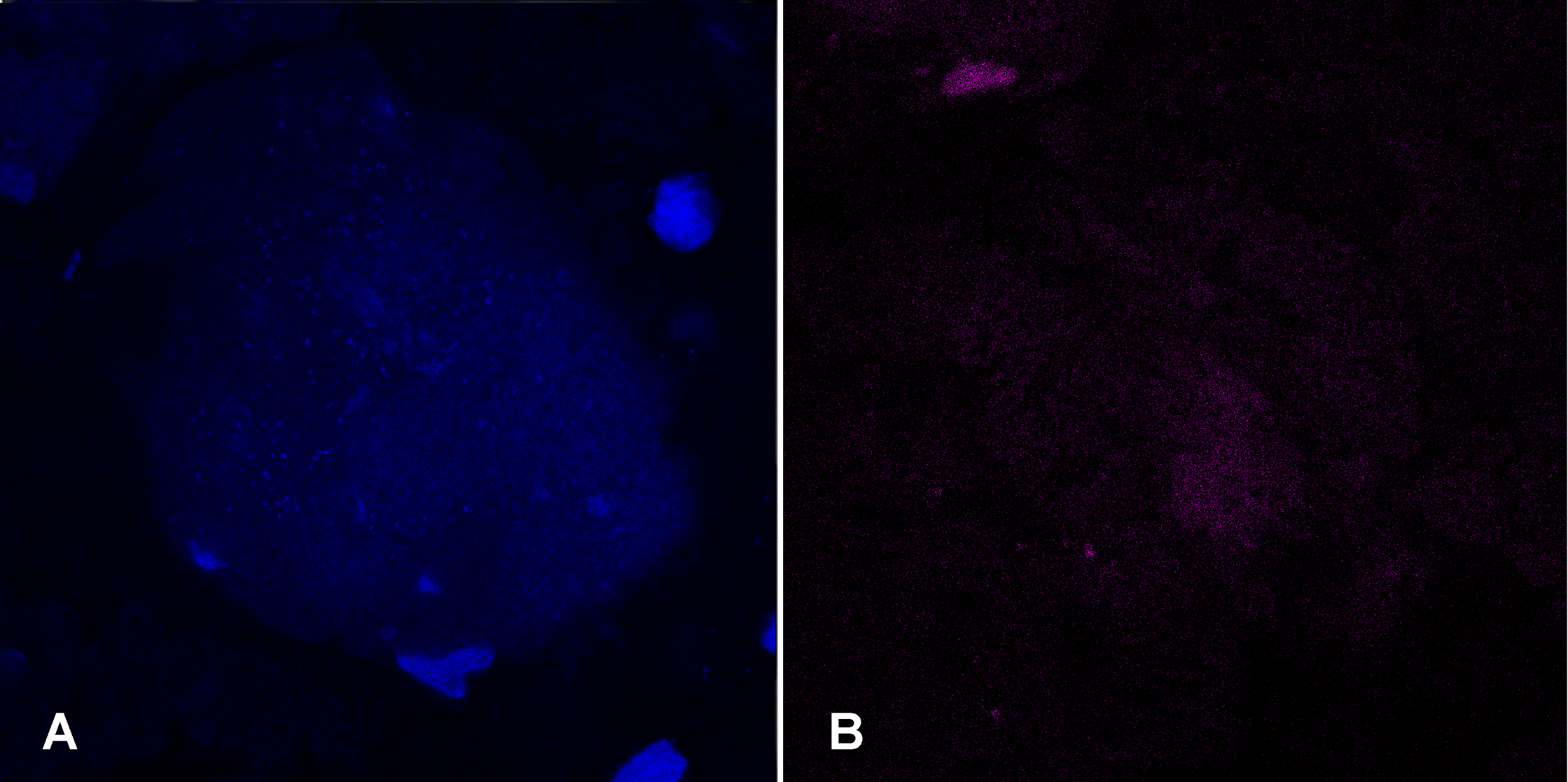

Supplement: S2 Fig — (A) Cellular nuclei stained for cell viability with DAPI (blue) and (B) negative signal after hybridisation with F. tularensis 23S rRNA probe labelled with the fluorochrome Cy5. (TIF) [file pone.0133593.s003.tif]

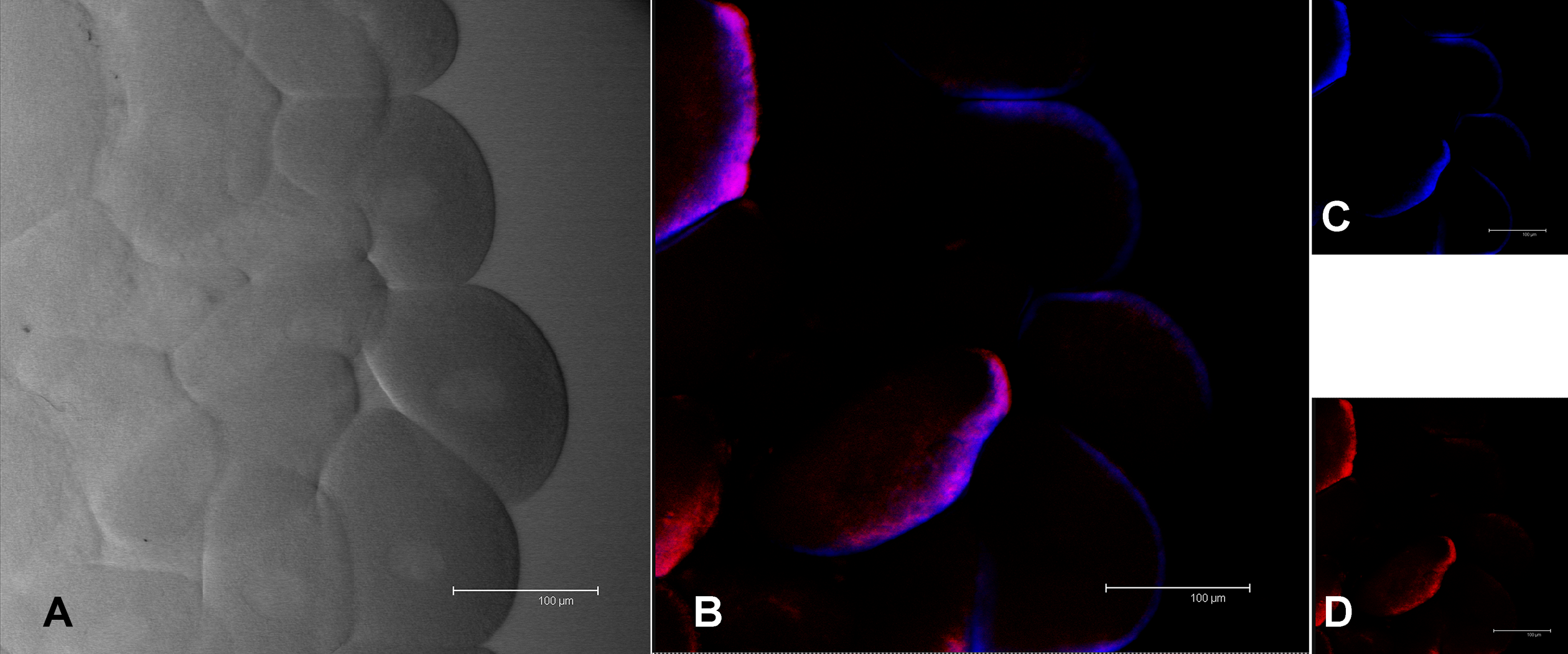

Supplement: S3 Fig — (A) Image obtained from light transmission; (B) Overlay image with blue signal for F. tularensis (23S rRNA probe for F. tularensis labelled with the fluorochrome Cy5) and red signal for universal eubacterial probe EUB338; (C) 23S rRNA probe for all F. tularensis; (D) universal eubacterial probe EUB338. Scale bar: 200 μm. (TIF) [file pone.0133593.s004.tif]

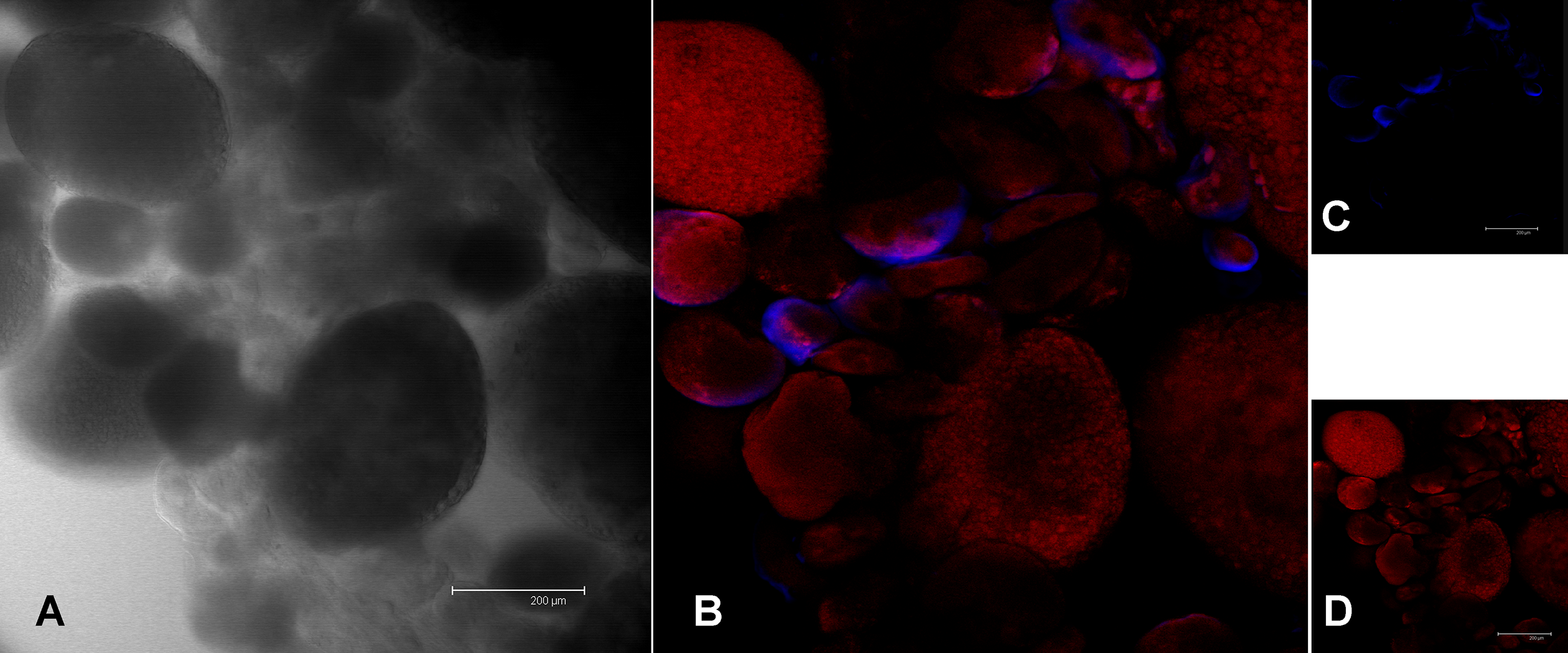

Supplement: S4 Fig — (A) Image obtained from light transmission; (B) Overlay image with blue signal for F. tularensis (23S rRNA probe for F. tularensis labelled with the fluorochrome Cy5) and red signal for universal eubacterial probe EUB338; (C) 23S rRNA probe for F. tularensis; (D) universal eubacterial probe EUB338. Scale bar: 100 μm. (TIF) [file pone.0133593.s005.tif]
